# Identification, functional characterization of a novel flavohemoglobin from *Ensifer adhaerens* and its application to enhancing vitamin B_12_ production

**DOI:** 10.1186/s12934-026-03073-z

**Published:** 2026-07-24

**Authors:** Jinmei Zhu, Shiyu Wang, Bo Li, Bolun Wang, Ping Wang, Jiequn Wu, Zejian Wang

**Affiliations:** 1 Collaborative Innovation Center of Yangtze River Delta Region Green Pharmaceuticals, Zhejiang University of Technology, Hangzhou, 310014 China; 2 State Key Laboratory of Bioreactor Engineering, East China University of Science and Technology, Shanghai, China

**Keywords:** Flavohemoglobin, *Ensifer adhaerens*, Cell growth, Vitamin B_12_

## Abstract

**Background:**

Bacterial flavohemoglobins are well-characterized as versatile proteins that significantly augment the biosynthesis yields of diverse value-added compounds in engineered microbial platforms. Our previous studies revealed marked upregulation of a flavohemoglobin-encoding gene in *Ensifer adhaerens* under oxygen-limiting conditions, with potential associations to vitamin B_12_ production.

**Results:**

In this study, a novel flavohemoglobin (FHP, encoded by *b12fla gene*) from *Ensifer adhaerens* were characterized. FHP exhibited high sequence and structural similarity to HMP (the flavohaemoglobin from *Halalkalibacterium halodurans*), yet demonstrating 3-fold higher NADH oxidase activity. Heterologous expression of FHP in *Escherichia coli* positively influences cell growth, biomass yield on carbon and nitrogen sources, increased OD_600_ and Oxygen Uptake Rate (OUR) in 50-L fermenter of FHP-expressing strain EcFHP (12% and 65% higher compared with control strain respectively). In addition, SEM analysis revealed that cells of overexpression strains exhibited obvious morphological alterations, manifesting as longitudinal extension or radial expansion compared to cells of wild-type strain. Overexpression of *b12fla* gene in *E. adhaerens* Casida A generated the recombinant strain Ea-b12fla, which produced 323 mg L^− 1^ of vitamin B_12_ in a 50-L fermentation while the wild-type strain yielded only 247 mg L^− 1^. Furthermore, cell growth, OUR, Carbon-dioxide Escape Rate (CER) and sugar utilization were obviously enhanced by overexpression of the flavohemoglobin.

**Conclusions:**

A flavohemoglobin from a new source was identified in this study. Heterologous expression in *E*. *coli* validated its ability to promote cell growth under specific oxygen supply conditions. Overexpression in *E. adhaerens* significantly enhanced cell growth and cell metabolism, while markedly increasing vitamin B_12_ production. Collectively, these findings demonstrate that flavohemoglobin (FHP) enhances cellular oxygen utilization while simultaneously improving the assimilation efficiency of carbon and nitrogen sources, and reveal the potential of flavohemoglobin for applications in high-density fermentation processes.

**Supplementary Information:**

The online version contains supplementary material available at https://doi.org/10.1186/s12934-026-03073-z.

## Introduction

High-cell-density fermentation of microorganisms is critical for sustainable biomanufacturing, particularly in the production of high-value bioproducts ‌such as‌ active pharmaceutical ingredients (APIs), biofuels, agrochemicals, ‌and‌ therapeutic proteins [1–4]. During high-cell-density fermentation of aerobic microorganisms, oxygen supply is an important parameter in aerobic fermentation since it is crucial in numerous intracellular biochemical reactions. However, oxygen transfer is inherently constrained due to the low O_2_ solubility in water, leading to inadequate supply, which becomes a critical bottleneck [5]. Air serves as the primary oxygen source in fermentation, typically supplied at 0.3–1.5 VVM. However, its low oxygen content (21%) necessitates dissolution into the broth and uniform dispersion as fine bubbles for optimal mass transfer to cells [6]. Traditional optimization of oxygen transfer rates (OTR) has long relied on several strategies: mechanical agitation, aeration rate modulation, oxygen enrichment and dissolved‑oxygen feedback control [7–10]. However, elevated agitation in stirred-tank bioreactors induces critical shear stress, triggering caspase-mediated apoptosis ‌or mechanical lysis in cells [11–13]. This necessitates balanced agitation-aeration optimization for industrial bioprocessing. The addition of pure oxygen or oxygen-enriched gas is generally costly, inefficient, and technically challenging, particularly in viscous fermentation broths [14–16]. Consequently, the development of cost-effective oxygenation methods represents a critical unmet need in industrial biotechnology to enhance fermentation efficiency.

The introduction and functional expression of hemoglobin (Hb) genes into diverse microorganisms and eukaryotes represents an effective genetic engineering strategy for improving oxygen transfer under oxygen-limited conditions. The hemoglobin from *Vitreoscilla* (VHb) has been characterized, because of its ability to transfer intracellular oxygen to help aerobic organisms adapt to low oxygen conditions [17, 18], was the first bacterial hemoglobin discovered and best-characterized member of the group of bacterial Hb proteins [19–23]. The *Vitreoscilla* hemoglobin has been successfully used to enhance microbial growth under oxygen-limited conditions or to boost the production of valuable compounds [24–29].

Flavohemoprotein and hemoglobin share high degree of sequence and structural similarity in their globin domain [30, 31]. In contrast to‌ *Vitreoscilla* hemoglobin (VHb), which comprises solely a heme-binding domain, flavohemoglobin (FHb) exhibits a dual-domain structure consisting of an N-terminal hemoglobin-like globin domain fused to a C-terminal flavin-binding domain [32]. The flavin domain resembles ferredoxin reductase, utilizing its inherent NAD(P)H oxidase activity to transfer electrons from NAD(P)H to the heme in the globin domain [33–35]. Flavohaemoglobins were first described in yeast as early as the 1970s, and they are ubiquitously distributed across bacterial and yeast species. The dual enzymatic roles as both reductases and oxidases of FHb present significant research challenges, while their physiological functions extend beyond those of hemoglobins [36]. Beyond its primary functions, FHb catalyzes the anaerobic conversion of nitric oxide (·NO) to nitrous oxide (N₂O), and generates deleterious superoxide (O₂⁻) via oxygen reduction in the absence of ·NO [37–41]. It is well established that overexpression of the hemoglobin gene not only stimulates overall microbial growth but also enhances the production of diverse biological molecules, including enzymes, amino acids, biofuels, and polymers, as well as secondary metabolites such as antibiotic. The overexpression of bacterial hemoglobin from *Sinorhizobium meliloti* (SHb) enhanced both cell growth and total fatty acid (TFA) content in *Mucor circinelloides* under both low and high aeration conditions [42]. The mutant strain of *Aureobasidium melanogenum* P16, which overexpresses the native flavohemoglobin gene, showed improvement in pullulan production and sugar utilization compared to the wild-type strain [43]. Engineered *Streptomyces lividans* TK24 with overexpress of hemoglobin SHb exhibits significantly enhanced secondary metabolite production compared to *Vitreoscilla* hemoglobin (VHb) transformants This metabolic improvement is mediated through SHb-dependent activation of the pentose phosphate pathway, which alleviates oxidative stress and elevates cellular NADPH levels. Heterologous expression of a *Cupriavidus necator* flavohemoprotein in *Escherichia coli* demonstrated a 50% enhancement in final cell density relative to *Vitreoscilla* hemoglobin (VHb)-expressing strains [44]. Expression of *Alcaligenes eutrophus* flavohemoprotein and engineered *Vitreoscilla* hemoglobin-reductase fusion protein improved hypoxic growth of *Escherichia coli* [45].

Cobalamin (vitamin B_12_) is an important nutrient for humans and animals with substantial global demand in pharmaceutical, food, and feed industries, serves as an essential coenzyme in mitochondrial/cytosolic pathways‌ (tricarboxylic acid cycle, one-carbon metabolism), methylation-mediated regulation‌ (metabolites, nucleic acids, proteins) and sex steroid modulation‌ through interactions between hosts and microbes [46–48]. The reported vitamin B_12_ producing natural or recombinant bacteria comprised genera *Pseudomonas shermanii*,* Acetobacterium*,* Bacillus*,* Corynebacterium*,* Escherichia*,* Flavobacterium*,* Methanobacillus*,* Pseudomonas*,* Streptococcus and Streptomyces* [49–51]. Nowdays The biomanufacturing of vitamin B_12_ primarily relies on fermentation-based production processes employing *Ensifer adhaerens* and *Pseudomonas denitrificans* as the predominant microbial chassis [52–54]. The network topology and metabolic fluxes of central carbon metabolism in the industrial vitamin B_12_ producing strain *Pseudomonas denitrificans* were characterized under oxygen limiting level, and the results demonstrated that oxygen levels impact the distribution of fluxes between the glycolysis pathway (EMP), Entner-Doudoroff pathway (ED), and Pentose Phosphate pathway (PPP) [55]. Furthermore, the successful implementation of a step-wise Oxygen Uptake Rate (OUR) control strategy revealed that dissolved oxygen levels regulate vitamin B₁₂ biosynthesis in *E. adhaerens* by modulating intracellular cofactor pools [56]. Under oxygen limitation, transcriptomic and metabolomic analyses of *E. adhaerens* revealed a marked upregulation of the *b12fla* gene over time, indicating that the bacterium enhances oxygen transport and storage by increasing expression of flavonhemoglobin (FHP). ‌Further knockout of the *b12fla* gene in *E. adhaerens* resulted in a significant reduction in VB_12_ production, demonstrating that the *b12fla* gene is a key gene of VB_12_ biosynthesis [57]. In the present study, the novel flavohemoglobin FHP was successfully characterized. The overexpression of *b12fla* was investigated to assess its impact on *E. coli* growth under oxygen-limiting conditions, as well as its effects on oxygen and nutrient utilization. Additionally, we then investigated the influences of *b12fla* overexpression on *E. adhaerens* growth and VB_12_ production.

## Materials and methods

### Strains, plasmids, and culture conditions of *E. coli*

The strains and plasmids used in this study are listed in Table S1, the primers are listed in Table S2. *E. coli* DH5α was used for cells competent for plasmid propagation were cultured in Luria-Bertani medium containing appropriate antibiotics at 37 °C with constant shaking. *E. coli* BL21(DE3) was used for overexpression of proteins. Luria-Bertani (LB) medium (10 g L^− 1^ tryptone, 10 g L^− 1^ NaCl, and 5 g L^− 1^ yeast extract) was used for *E. coli* activation. M9 medium (3 g L^− 1^ KH_2_PO_4_, 1 g L^− 1^ NH_4_Cl, 0.5 g L^− 1^ NaCl, 6.78 g L^− 1^ Na_2_HPO_4_, 0.246 g L^− 1^ MgSO_4_·7H_2_O, 44.1 mg L^− 1^ CaCl_2_·2H_2_O, 3 g L^− 1^ glucose) was employed for shake flask fermentation. Terrific Broth (TB) medium (12 g L^− 1^ tryptone, 24 g L^− 1^ yeast extract, 5 g L^− 1^ glycerol, 2.31 g L^− 1^ KH_2_PO_4_, 12.54 g L^− 1^ K_2_HPO_4_) was used for protein expression.When needed, appropriate antibiotics (50 mg L^− 1^ kanamycin, 50 mg L^− 1^ apramycin) were added to the culture medium.

### In silico modeling, docking and MD simulations

The simulated three-dimensional (3D) structures of proteins were predicted from the AlphaFold2 Web server (https://github.com/deepmind/alphafold AlphaFold2). The accuracy of models was checked by SAVES v6.0 Web site (https://saves.mbi.ucla.edu). All structures were prepared for analysis by removing ligands and water molecules. The Discovery Studio Client 2019 was used for a protein structure similarity comparison. then was used to dock the staring structure of the FHP in complex with the co-factor heme. The initial structures were created by PyMOL (http://www.pymol.org).

### Overexpression of FHP, VHb and HPM in *E. coli* and purification

The *vgb* and *b12fla* gene were amplified from *Vitreoscilla* sp. C1 and *E. adhaerens* Casida A by the PCR using gene specific primers in Table S1. The *hmpBh* gene from *Halalkalibacterium halodurans* was synthesized by Beijing Tsingke Biotech Co. Ltd. Sequence of genes and proteins were shown in Table S3. These three genes were ligated into plasmid pET28a between *Nco*Ⅰ and *Hind*Ⅲ restriction sites to obtain overexpression plasmids pET28a-*vgb*, pET28a-*b12fla* and pET28a-*hmpBh*. Overexpression strains EcFHP, EcVHb, and EcHMP were generated by transforming recombinant plasmids into *E*. *coli* BL21(DE3). The recombinant proteins were purified using affinity chromatography using a nickel resin (Tiandirenhe, Changzhou, China), in the buffer with 20 mM Tris-HCl (pH 7.5) and 250 mM NaCl at 4 ℃. The pured proteins were quantified by Bradford method [58] and resolved on 12% SDS-PAGE.

### Identification of flavin cofactors bound to FHP

For flavin cofactors analysis, 40 µL purified FHP solution (20.6 mg mL^− 1^) was supplemented into 960 µL of 100 mM Tris-HCl buffer (pH 7.5). The mixture was incubated in a boiling water bath at 100 °C for 10 min to denature the target protein and release bound flavin cofactors. Denatured precipitated proteins were removed by centrifugation at 12,000×g for 15 min at 4 °C followed by filtration through a 0.22 μm microporous filter. The resulting supernatant containing free flavins was subjected to ultraviolet-visible (UV-Vis) absorbance spectrum scanning from 190 nm to 500 nm using a UV-1800 UV spectrophotometer (SHIMADZU, Japan).

### Construction of chromosomally integrated strains

Using the pCas/pTarget system (also designated as pEcCas/pEcgRNA) [59], plasmids pEcgRNA-sg^recA^-L^recA^-*b12fla*- R^recA^ and pEcgRNA-sg^ara^-L^ara^-*b12fla*- R^ara^ were constructed for integrating the T7-*b12fla* expression cassette into the recA and ara site, respectively, followed by the generation of integrated overexpression strains EcFHP-ΔrecA and EcFHP-ara. Sequence of sgRNAs were designed to target sequences near 5′ and 3′ ends of the replacement region in the *E. coli* BL21(DE3) chromosome. Sg^recA^ and sg^ara^ targeted the recA region and ara region respectively (Table. S1). sgRNA oligos were cloned into plasmid pEcgRNA as previously described (Table. S1).

### Enzyme activity assays and enzyme kinetic parameter measurement

The determination of NADH oxidation activity was performed in typical reaction (1000 µL) includes 100 mM PBS buffer (pH 7.5) and 0.5 mg mL^− 1^ purified HMP or 0.2 mg mL^− 1^ purified FHP, 100 µM NADH. NADH oxidation activity were measured by a UV-1800 UV spectrophotometer (SHIMADZU, Japan) based on monitoring the decrease of NADH at 340 nm (ε = 6220 L·mol^− 1^·cm^− 1^) at 30 °C in 1 mL quartz cuvettes. One unit of activity was defined as the amount of enzyme that catalyzed the oxidation of 1 µmoL NADH per minute under the optimal condition. All activities were measured in triplicates and error bars indicate the standard deviation. The protein concentration was quantified by the Bradford method using bovine serum albumin (BSA) as the standard [60].

Initial velocities at various concentrations of the substrate NADH (0.03–0.1 mM) were measured at 30 °C to obtain the apparent *K*_m_ values of the purified HMP or FHP. For activity assay to calculate kinetic parameters, the cofactor of NADH at saturated concentration towards the enzyme was added in the reaction mixture. The kinetic parameters were further determined from Michaelis-Menten equation.All activities were measured in triplicates and error bars indicate the standard deviation.

### Cultivation of *E. coli* in shack-flask

Single colonies of *E. coli* strains from the plate were inoculated into 5 mL of LB medium, and cultured overnight at 37 °C with shaking at 220 rpm. The resulting cell suspension was then inoculated into 20 mL of M9 medium with 1% inoculum. when the bacterial culture attained an optical density at 600 nm (OD₆₀₀) of approximately 0.6, protein expression was induced by the addition of 0.1 mM isopropyl β-D-1-thiogalactopyranoside (IPTG) into the media. Shake-flask fermentation was performed at 37 °C and 220 rpm.

### Cultivation of *E. coli* in 50-L bioreactor

Single colonies of *E. coli* strains from the plate were inoculated into 5 mL of LB medium (primary seed culture) containing 50 µg mL^− 1^ kanamycin and incubated at 37 °C with shaking at 220 rpm for 10–12 h. The primary seed culture was then inoculated into 50 mL of LB medium (in 500 mL shake flask), after culture for 10–12 h and then inoculated into 30 L TB medium (in 50 L bioreactor). For fermenter cultivation, initial conditions included an agitation speed of 500 rpm, an aeration rate of 1.0 vvm, and a temperature of 37 ℃. During fermentation, dissolved oxygen was controlled at ≥ 30% by adjusting agitation speed, pH was maintained at approximately 6.5 using ammonia solution, and glucose concentration was regulated at 1–2 g L^− 1^ by feeding 600 g L^− 1^ glucose. The culture was grown for approximately 24 h. Throughout the fermentation process, pH, dissolved oxygen, and off-gas data were monitored and recorded using Biostar software.

### Determination of OD_600_, OD_700,_ nitrogen, and glucose in the medium

The optical density (OD) of the fermentation broth at 600–700 nm was measured using a SYNERGY-LX microplate reader (BioTek, USA). The fermentation culture was separated via centrifugation to isolate the cells and supernatant. The glucose concentration in the supernatant was spectrophotometrically determined using the 3,5-dinitrosalicylic acid (DNS) reagent a SYNERGY-LX microplate reader (BioTek, USA) at 540 nm, while the ammonia nitrogen concentration was measured using Nessler’s reagent through a colorimetric assay using a SYNERGY-LX microplate reader (BioTek, USA) at 420 nm. Biomass yield coefficient per glucose [Y_x/glc_, g^− 1^] was calculated according to previously reported [61].

### Construction of *E. adhaerens* strain expressing *b12f*la gene

The strain Ea-b12fla was constructed for overexpression of *b12f*la. We amplified sequence of promoter ProP (promoter of ORF WFP91548.1) and the *b12fla* from the genome of *Ensifer adhaerens* Casida A with the primer pair ProP-F/ProP-R and 260-*b12fla*-F/260-*b12fla*-R respectively. The fragments was ligated into plasmid pOJ260 between *Eco*RⅠ and *Hin*dⅢ restriction sites, to obtain the overexpression plasmid pOJ260-*b12f*la. Then, the suppression and overexpression plasmids were introduced into *Ensifer adhaerens* by intergeneric conjugation using *E. coli* S17-1. The colonies that were apramycin resistant were identified as recombinant strains, PCR and DNA sequencing were used to confirm the genotypes further.

### Fermentation of *E. adhaerens*

For fermentation in a bioreactor, a certain amount of the plate-cultured strain was inoculated into seed flask with a working volume of 100 mL, and cultivated at 260 rpm and 32 °C until the optical density (OD_700_) of the culture reached 13–15. The seed medium consisted of 40 g L^− 1^ sucrose, 20 g L^− 1^ corn steep liquor, 5 g L^− 1^ betaine, 1 g L^− 1^ (NH_4_)_2_SO_4_, 2 g L^− 1^ (NH_4_)_2_HPO_4_, 0.8 g L^− 1^ MnSO_4_, 0.02 g L^− 1^ CoCl_2_, 0.3 g L^− 1^ MgO, 0.01 g L^− 1^ DMBI (5,6-dimethylbenzimidazole), 0.01 g L^− 1^ ZnSO_4_, and 1.5 g L^− 1^ CaCO_3_. The well-cultured seed was then inoculated into a 50 L bioreactor. The fermentation conditions were set at 32 °C, 400 rpm, and the aeration rate of 1vvm. The residual sugar and betaine concentration were maintained at 30 ± 4 g L^− 1^ and 5 ± 1.5 g L^− 1^ through continuous feeding glucose and betaine media, respectively. The fermentation medium consisted of 80 g L^− 1^ sucrose, 60 g L^− 1^ corn steep liquor, 28 g L^− 1^ betaine, 1 g L^− 1^ (NH_4_)_2_SO_4_, 0.75 g L^− 1^ (NH_4_)_2_HPO_4_, 0.075 g L^− 1^ CoCl_2_, 0.5 g L^− 1^ MgO, 0.05 g L^− 1^ DMBI (5,6-Dimethylbenzimidazole), 0.08 g L^− 1^ ZnSO_4_, and 1 g L^− 1^ CaCO_3_. For fermentation in shake flask, the seed medium and fermentation medium were the same as fermentation in a bioreactor.

### Scanning electron microscopy (SEM)

Cells were first fixed with 2.5% (v/v) glutaraldehyde for more than 4 h and then washed with 0.1 M phosphate-buffered saline (PBS) (pH 7.3) (three times, 10 min each). The fixed cells were washed by ethanol in concentration gradient (v/v) of 30, 50, 70, 80%, 90%, and 100% in a sequential way and further dehydrated by tert- butanol mixed with ethanol in a ratio of 1:1. The sample was treated with pure tert- butanol and then used for scanning electron microscopy (HITACHI S-4700, JAPAN) imaging after lyophilization.

## Results and discussion

### Identification and molecular modeling analysis of FHP

**Fig. 1:**
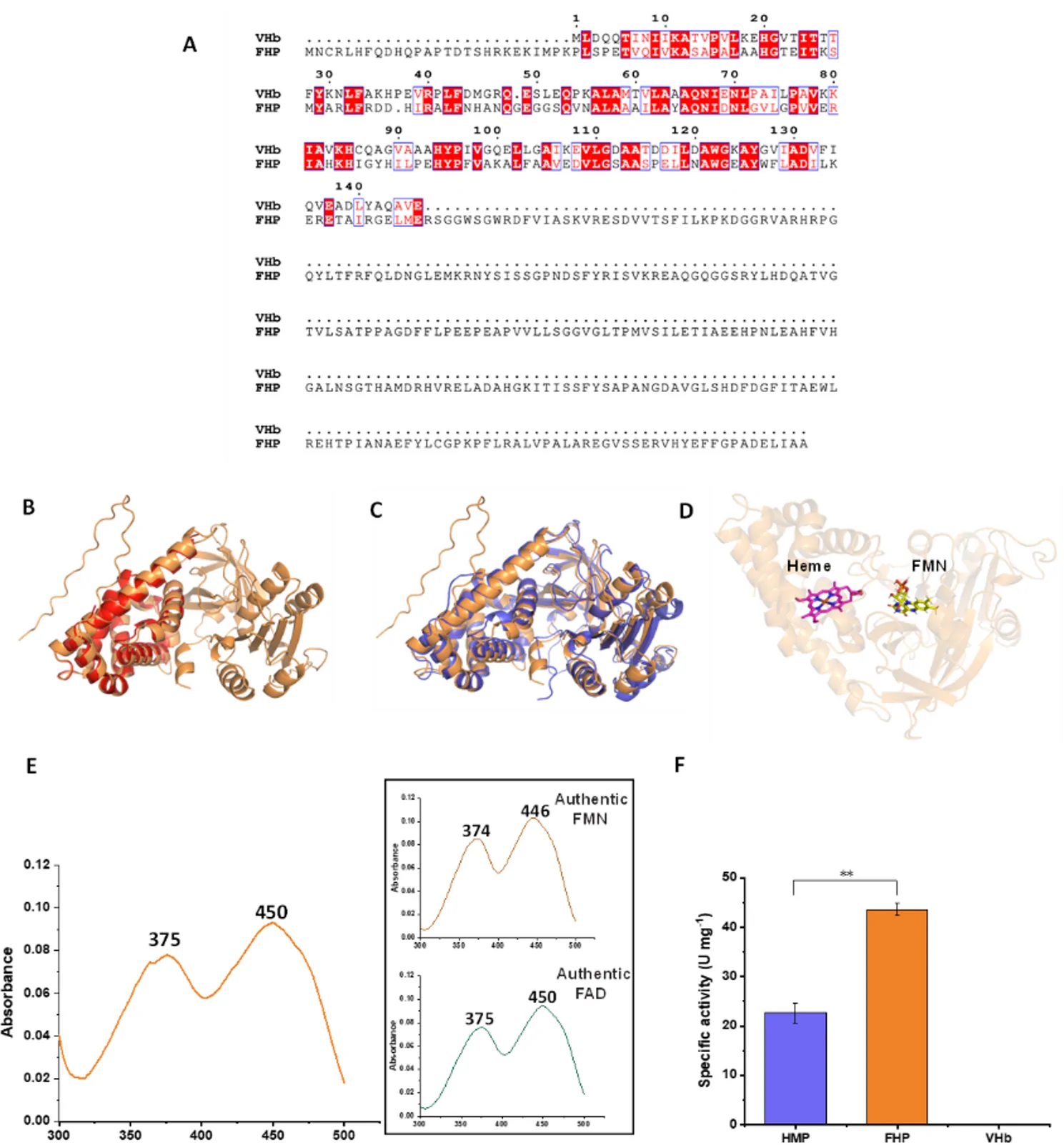
Molecular modeling analysis, expression and NAD(P)H oxidase activity of FHP. The simulated structures of FHP, HMP and VHb are modeled using AlphaFold2 Web server. **A** Amino acid sequence alignment of FHP and VHb. **B** Alignment of structure of FHP and VHb. The structure of FHP was shown as orange while structure of VHb was shown as red **C** Alignment of structure of FHP and HMP. The structure of FHP was shown as orange while structure of HMP was shown as violet-blue **D** In silico model of FHP with heme and FMN. The simulated structure of FHP was docked with heme (magenta) and FMN (yellow) **E** Absorption spectrum for released cofactor from FHP. The absorbance peaks at ~ 375 and ~ 450 nm is diagnostic of a flavoprotein. The inset shows the absorption spectra for authentic FMN (top) and FAD (bottom). **F** NADH oxidase activity of purified FHP, VHb and HMP. The NADH oxidative activity was measured using spectrophotometric method by monitoring change of characteristic absorbance of NADH at 340 nm

A novel hemoglobin from *E. adhaerens* Casida A was identified. The CDS of FHP was 1287 bp (Fig. 1A), encoding 429 amino acids with predicted protein molecular weights of 46.9 kDa. Using the FHP sequence as a query against the NCBI database, we identified 1000 flavohemoglobin enzyme sequences (Fig S1). Highly similar sequences were eliminated to minimize redundancy, following the construction of a phylogenetic tree, phylogenetic analysis revealed that FHP is phylogenetically distant from most previously reported and characterized flavohemoglobins. Notably, the flavohemoglobin ‌SHb‌ from *Sinorhizobium meliloti* has been identified and functionally confirmed to possess the biological functions of promoting cell growth and enhancing fatty acid biosynthesis, it clusters within a separate clade far from the FHP-containing branch. FHP may represent an uncharacterized member of the flavohemoglobin family, which confers important research value to investigate its biochemical function and influences on cellular physiological metabolism.

Flavohaemoglobin from *Halalkalibacterium halodurans* (HMP) has been previously reported to enhance microbial growth phenotypes, as demonstrated by HMP-expressing cells achieving 25% higher final optical densities than VHb-expressing cells, respectively, along with an obvious increase in biomass yield on glucose [61]. Consequently, this protein was selected for structural and functional comparisons with FHP. Multiple sequence alignment of three proteins shows that FHP has 40% identity to the sequences of VHb and 43% identity to HMP from *H. halodurans*, respectively. Consistent with the structure of flavohemoglobin HMP, FHP possesses three conserved domains, the N-terminal globin domain with heme binding site between positions 1 and 175, the C terminal redox enzyme domains with NAD-binding site and redox enzyme domain flavin cofactor-binding site between positions 176 and 428 (Fig. 1A).

Superimposition of simulated three-dimensional structures of FHP, VHb and HMP further revealed high structural similarity between globin domain of FHP and VHb (Fig. 1B), while FHP contained additional highly-conserved flavin cofactor and NAD(P)H-binding domain. FHP and HMP exhibited significant structural overlap (Fig. 1C), Thus, to explore structural/functional compatibility between FHP and heme cofactors, heme was docked into globin domain of FHP. The results revealed that heme can be docked at positions that are favorable for its interaction with the active site of FHP (Fig. 1D). Further spectroscopic analyses were performed to verify the presence of the heme cofactor. The results showed that the free heme exhibited a typical broad soret band at ~ 380 nm, while the absorbance maximum of the flavohemoglobin FHP was located at ~ 405 nm in PBS buffer. This absorbance maximum of FHP is consistent with the previously reported value of ~ 405 nm for hemoglobin in PBS [62], which confirms the heme cofactor bound to FHP (Fig S2). The flavin cofactor bound to FHP was identified using the spectroscopic method. Authentic flavin adenine dinucleotide (FAD) and flavin mononucleotide (FMN) were used as controls. The absorption spectrum of released cofactor had absorption maxima at ~ 375 and ~ 450 nm (Fig. 1E), given the high similarity between the UV-visible spectra of FAD and FMN, the flavin cofactor bound to FHP was further analyzed via high-performance liquid chromatography (HPLC). The HPLC results confirmed that the cofactor associated with FHP was FMN, while the cofactor bound to HMP was identified as FAD (Fig S3). The modeling analysis and identification of flavin cofactor suggesting that FHP may possess functions exclusive to flavohemoglobin, such as resistance to nitrosative stress, NAD(P)H-dependent enzyme activity and scavenging of reactive oxygen and nitrogen species which are absent in conventional hemoglobins.

### Protein expression and NADH oxidase activity of recombinant FHP

Previous studies of flavohemoglobin have shown that they have the ability to oxidise NADH and consume oxygen. Flavohemoglobin FHP contains one heme and one FMN as prosthetic groups, these observations imply a potential capacity for NADH oxidation, suggesting its functional convergence with canonical NADH-oxidizing enzymes. To determine whether flavohemoglobin from *E. adhaerens* process NADH oxidation activity, the recombinant FHP was overexpressed in *E. coli* and purified using the nickel affinity chromatography using a nickel resin. Meanwhile, flavohemoglobins VHb and HMP were expressed and purified. SDS-PAGE analysis shown the presence of the FHP, VHb and HMP proteins were about 47.7 kDa, 16.6 kDa and 48.2 kDa (Fig S4).

The NADH oxidative activity of FHP, VHb and HMP were investigated using spectrophotometric method by monitoring change of characteristic absorbance of NADH at 340 nm. The results demonstrated that VHb has no NADH oxidation activity, due to the absence of flavin-binding domain which is the critical functional module in flavohemoglobins for catalyzing NADH oxidation. While both FHP and HMP exhibited obvious NADH oxygenase activity (Fig. 1F). The novel flavohemoglobin FHP exhibits about 2-fold higher NADH oxidase activity when the NADH concentration is 0.1 mM (with specific activity 43.64 U mg^−1^) compared to that of HMP (with specific activity 22.68 U mg^−1^). Further analysis of the kinetic parameters revealed that HMP showed lower *K*_*m*_ to NADH.The results indicate that compared with HMP, FHP has a lower binding affinity for NADH. However, FHP exhibits an approximately 2-fold higher *k*_*cat*_ value toward NADH than HMP, which endows FHP with about 1.5-fold higher catalytic efficiency than HMP (Fig. S5).

### Effects of FHP expression on cell growth of *E. coli*

**Fig. 2:**
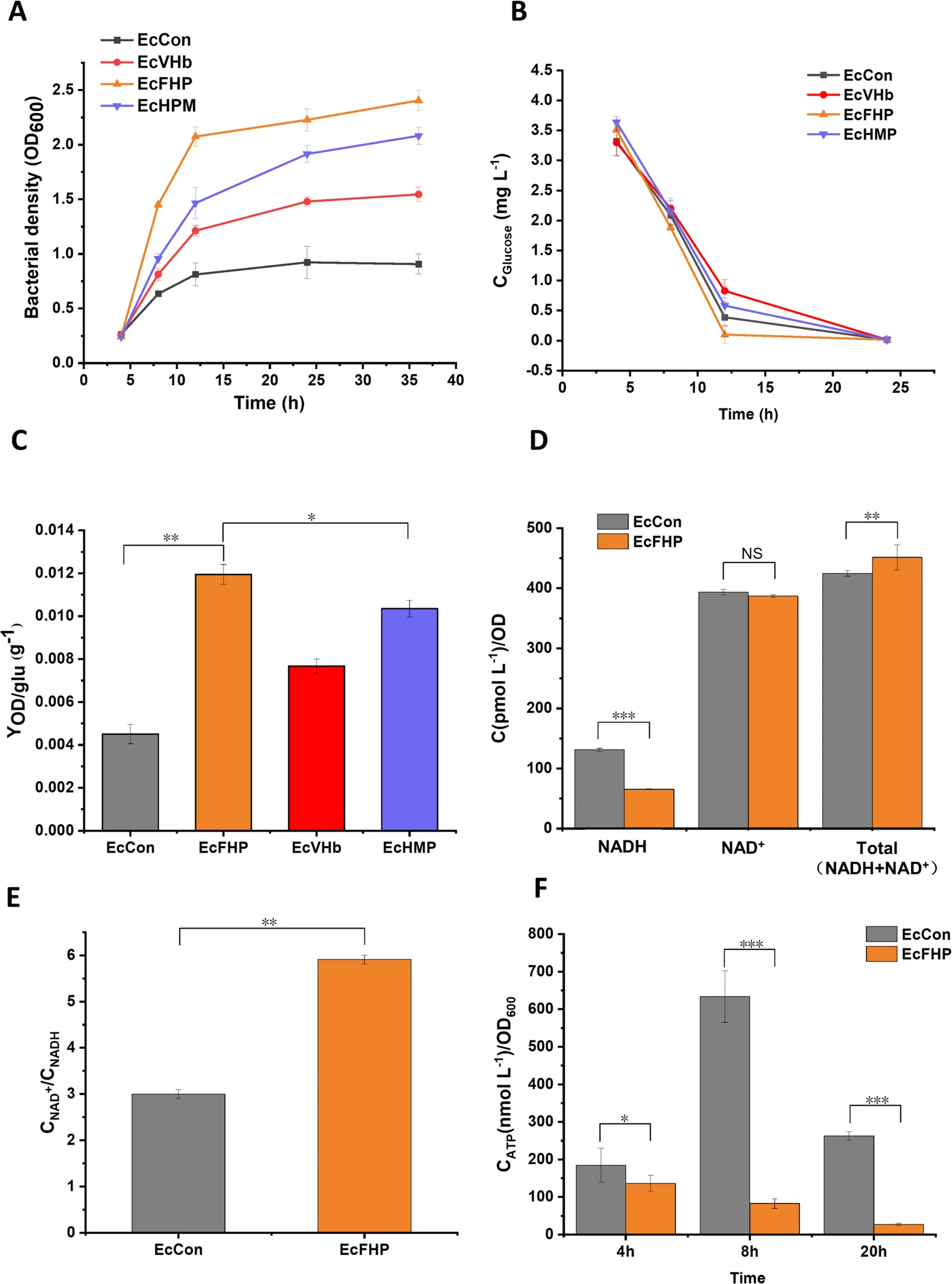
Effects of flavohemoglobins expression on growth and cellular metabolism of *E. coli*. The plasmid-bearing strains EcFHP (overexpressing the *b12fla* gene), EcVHb (overexpressing the *vgb* gene), EcHPM (overexpressing the *hmpBh* gene) and the control strain EcCon (bearing plasmid pET28a) were cultured in M9 or LB medium at 37 ℃ at flask level. **A** The time course of biomass of strain EcFHP, EcVHb, EcHPM and EcCon. **B** Residual glucose concentrations. **C **Yield of biomass on glucose (Y_OD/glc_, g^− 1^), **D** NADH, NAD^+^ concentrations of strain EcFHP and EcCon. **E** NAD^+^/NADH of strain EcFHP and EcCon. **F** ATP concentrations of strain EcFHP and EcCon. The inducer IPTG was added to the culture after 4 h of incubation. Data are presented as mean values of three biological replicates ± standard deviation. Asterisks depict significant differences between samples: *p < 0.05, **p < 0.01 and ***p < 0.001

To assess the effects of FHP, VHb and HMP heterologous overexpression on the cell growth and utilization of glucose and ammonia nitrogen of *E. coli*. The previously constructed *E. coli* mutant strains EcFHP strain (bearing plasmid pET28a-*b12fla*), EcVHb strain (bearing plasmid pET28a-*vgb*) and EcHMP strain (bearing plasmid pET28a-*hmpBh*), as well as control strain EcCon (bearing plasmid pET28a) were cultivated in flasks in M9 medium with glucose as a carbon source and induced by addition of IPTG (plasmids can be found in Table S1). The OD_600_ values, residual glucose and ammonia concentrations were determined (Fig. 2). The growth of strains was monitored by measuring OD_600_, the measurements revealed that growth of over-expressing strains was significantly better than that of the control strain EcCon. With addition of IPTG, strains expressing hemoglobin or flavohemoglobin initiated accelerated growth compared to the control strain. Strain EcFHP exhibiting significantly faster growth than strains EcVHb and EcHPM. After cultured for about 12 h, the growth of the all strains entered the stationary phase, accompanied by an obvious slowdown in growth rate (Fig. 2A). The over-expressing strains demonstrated substantially higher final OD_600_ values than EcCon at the end of 36 h cultivation, EcFHP strain cells had the highest final OD_600_ values, which were 2.6-fold higher than the control strain, 1.5-fold higher than EcVHb, and 1.15-fold superior to EcHMP, respectively (Fig. 2A). To confirm that the increase in culture OD was due to an elevation in cell number rather than alterations in cellular morphology, bacterial cell counts for both the control strain EcCon and the over-expressing strain EcFHP were determined by flow cytometry. The results revealed that strain EcFHP exhibited a 5.28-fold higher in cell number compared to the wild-type strain (Fig. S6). Our results suggest that under the same oxygen supply condition, FHP exhibits superior growth-promoting efficacy compared to VHb and HMP.

### Effects of FHP expression on cellular metabolism of *E. coli*

Residual glucose concentration was measured to monitor the rate of glucose consumption in strain EcFHP, EcVHb, EcHMP, and EcCon. The glucose consumption rate of strain EcFHP was slightly faster than the other three strains. After cultured for about 24 h, glucose in the culture medium was nearly zero in all strains, indicating that all available glucose had been consumed completely (Fig. 2B). The yields of biomass on glucose (Y_OD/glc_, g^− 1^) were calculated based on the OD_600_ and final glucose consumption of each strain. The results demonstrated that strains overexpression FHP, VHb and HPM exhibite significantly higher glucose utilization compared to the control strain. Notably, the glucose utilization rate of EcFHP reached approximately 0.012 g^− 1^, which was 1.15-fold and 1.55-fold higher than those of EcHMP and EcVHb, respectively (Fig. 2C). The results suggest that FHP outperforms HMP and VHb in promoting carbon source utilization to promote cell growth.

The contents of NADH and NAD⁺ were determined in the EcCon and EcFHP strains. The results showed that there was no significant difference in NAD⁺ content in EcFHP compared with the control strain; however, the NADH content in EcFHP was substantially lower than that in the control strain, reaching only approximately half of the level observed in EcCon (Fig. 2D). We speculate that this is due to the NADH oxidase activity of FHP, which oxidizes NADH to NAD⁺ in the FHP-overexpressing strain, thereby perturbing the NAD⁺/NADH balance. Indeed, the NAD⁺/NADH ratio in the EcFHP strain was found to be approximately two-thirds lower than that in the EcCon strain (Fig. 2E). Furthermore, determination of ATP content in the EcCon and EcFHP strains revealed that the ATP level in EcFHP was markedly lower than that in EcCon. Taken together with the OD values and glucose consumption data, we propose that overexpression of FHP promotes cellular sugar utilization, which is primarily directed toward energy-consuming anabolic processes.

Furthermore, residual ammonia nitrogen in the culture supernatant was measured. After 12 h, the ammonia concentration in culture media all of all strains hardly decreased further, indicating that ammonia nitrogen consumption had almost stopped (Fig. S7). Yield of biomass on ammonia nitrogen (Y_OD/NH₃−N_, g^− 1^) was calculated using OD_600_ and final ammonia nitrogen consumption. Compared to the control, EcFHP achieved a highest ammonia nitrogen utilization than both EcHMP and EcVHb (Fig. S8), demonstrating that FHP enhances nitrogen source utilization more effectively than HMP or VHb, thereby facilitating faster bacterial growth and proliferation.

**Fig. 3:**
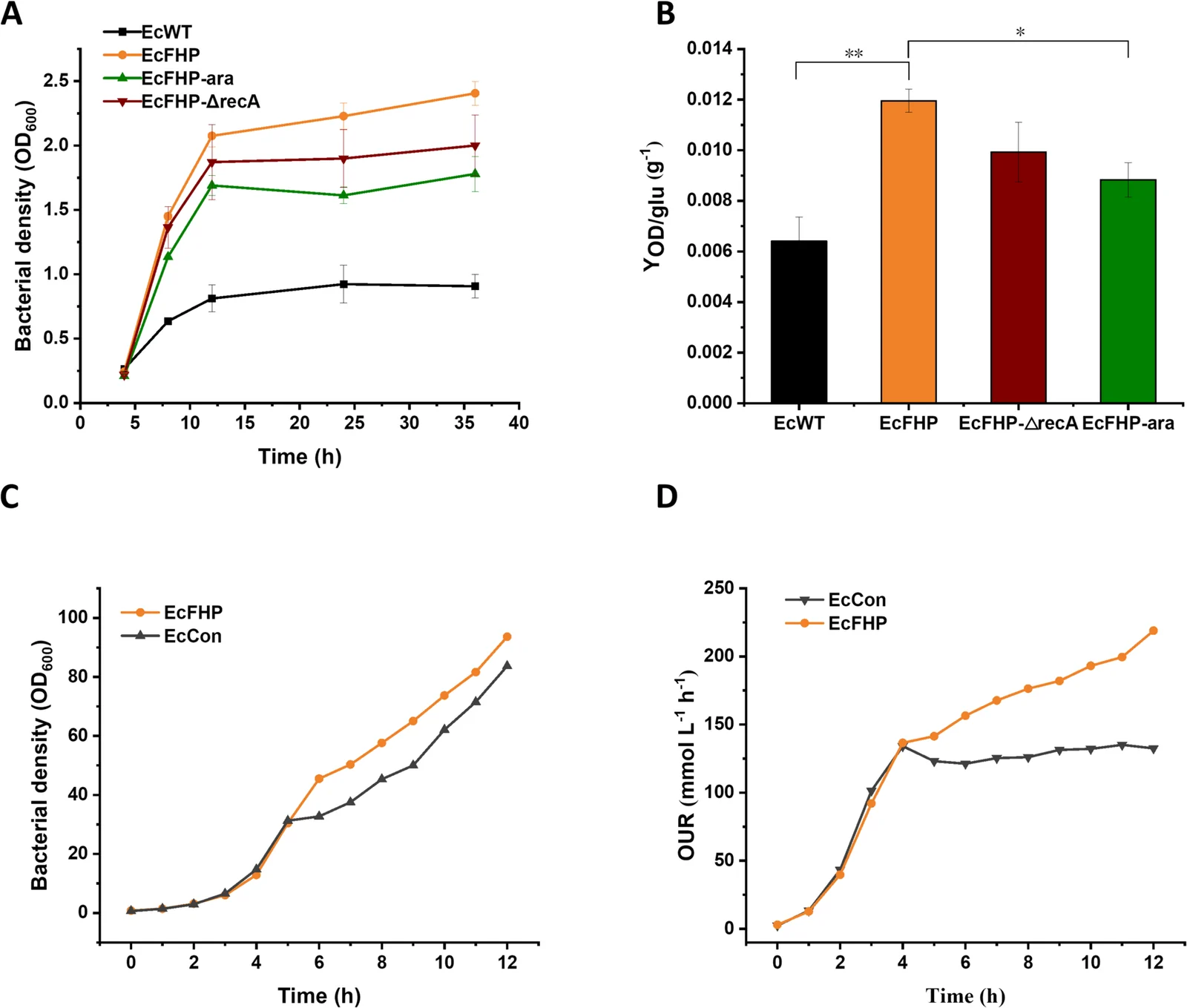
Growth, utilization of glucose of integrated strains and Effects of FHP expression on cell metabolism of *E. coli* in bioreactor. The chromosomally integrated FHP expression strains EcFHP-ΔrecA (integrated at the recA site) and EcFHP-ara (integrated at the ara site) were cultured in M9 medium at 37 ℃ at flask level. **A** The time course of biomass of strain EcFHP, EcVHb, EcHPM and EcCon. **B** Yield of biomass on glucose (Y_OD/glc_, g^− 1^) and **C** The time course of biomass and **D** OUR of EcCon and EcFHP strain cultivated in 50-L bioreactor. The inducer IPTG was added to the culture after 4 h of incubation. Data are presented as mean values of three biological replicates ± standard deviation. Asterisks depict significant differences between samples: *p < 0.05, **p < 0.01 and ***p < 0.001

To obtain more manageable engineered strains, the *b12fla* gene was integrated into the *E. coli* genome via site-specific recombination. recA (encoding recombinase A) and the arabinose operon (ara) region were selected as integration sites to evaluate the impact of chromosomal location on gene expression. Two chromosomally integrated strains, EcFHP-ΔrecA and EcFHP-ara, were successfully constructed and cultivated in shake flasks. The results revealed that both integrated strains exhibited slower cell growth compared to the plasmid-based overexpression strain ‌EcFHP, and the final OD_600_ value of strain EcFHP was 20% and 30% higher relative to EcFHP-ΔrecA and EcFHP-ara, respectively (3 A). Furthermore, the yields of biomass on glucose of EcFHP was markedly higher than that of the chromosomally integrated strains (Fig. 3B). We hypothesize that *b12fla* gene overexpression via episomal plasmid and genomic integration influences the final protein expression levels, and thereby affects the degree of enhancement in cellular growth and carbon source utilization.

### Effects of FHP expression on cell metabolism of *E. coli*

We further evaluated growth and oxygen consumption of the strain EcFHP and the control strain EcCon in 50-L bioreactors. Before induction, both strains exhibited comparable biomass accumulation (OD_600_) and oxygen uptake rate (OURs). However, with the addition of inducers at the fourth hour, the *b12fla*-overexpressing strain EcFHP showed a significantly increased growth rate than the control strain. The *b12fla*-overexpressing strain exhibited an 11.9% higher biomass accumulation (OD_600_) than the control strain (Fig. 3C). The oxygen uptake rate (OUR) of EcFHP strain progressively increased with the addition of IPTG, while that of EcCon strain remained essentially stable. By 12 h, the OUR of EcFHP strain reached 218.96 mmol L^− 1^ h^− 1^, representing a 65% enhancement compared to that of the control strain (132.50 mmol L^− 1^ h^− 1^) (Fig. 3D). The results indicating that overexpression of flavohemoglobin FHP enhances both cellular growth and oxygen uptake capacity of *E. coli*.

### Morphology changes stimulated by overexpression of *b12fla*

**Fig. 4:**
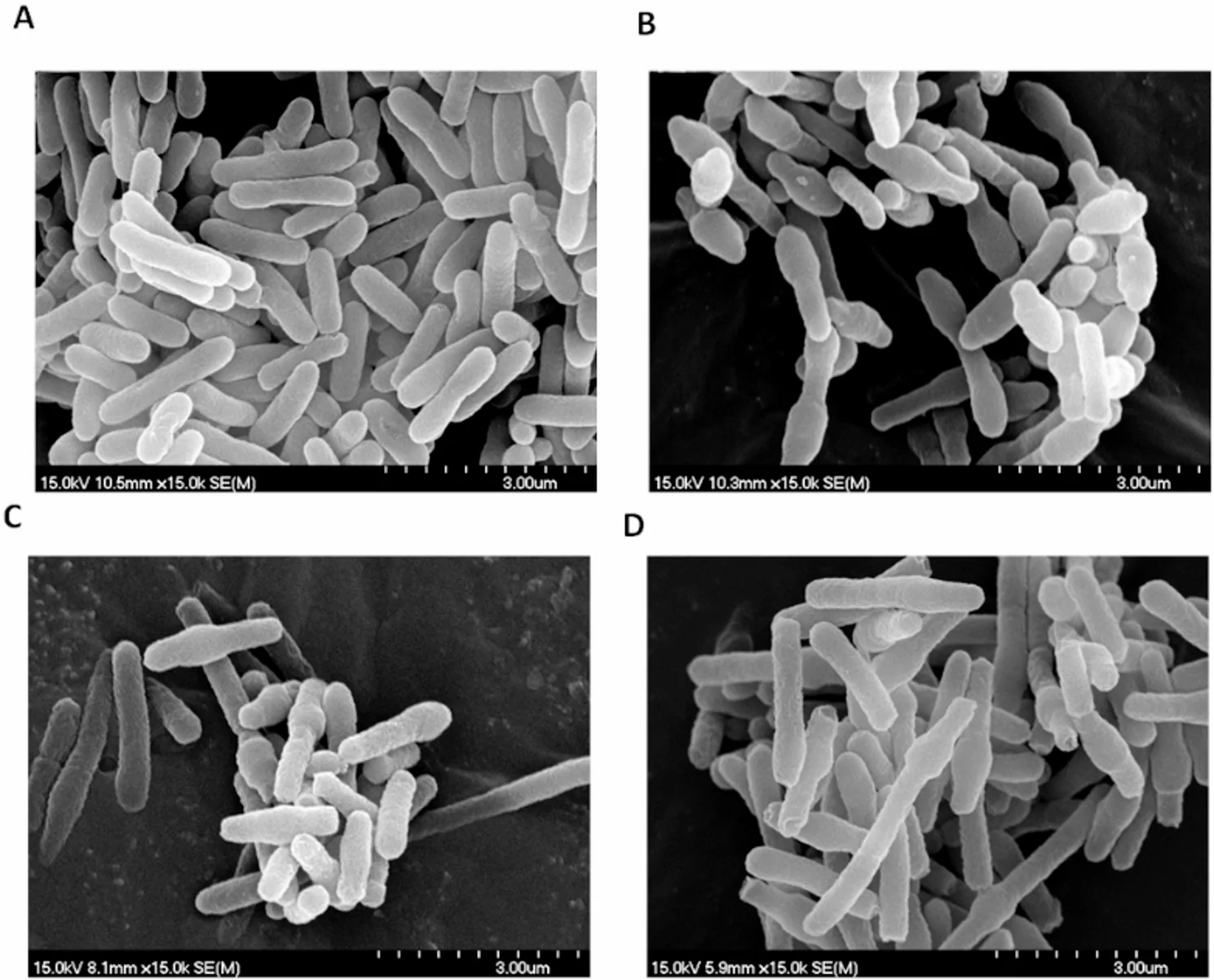
Scanning electron microscopy studies on morphology of EcCon (**A**), EcFHP (**B**), EcFHP-ΔrecA (**C**) and EcFHP-ara (**D**). The plasmid-bearing strains EcFHP (overexpressing the b12fla gene), the control strain EcCon (bearing plasmid pET28a), and chromosomally integrated FHP expression strains EcFHP-ΔrecA (integrated at the recA site) and EcFHP-ara (integrated at the ara site) were cultured in a TB medium at 37 °C. FHP-overexpressing strains exhibited morphological alterations, genome-integrated strains displayed distinct morphology changes with the plasmid-bearing overexpression strain (more pronounced cell elongation)

Cell shape and dimension has significant effects on cellular growth patterns and metabolic functions [63–65]. The diverse shapes and morphology of cells are maintained by protein cytoskeletal and morphological elements, such as actin, tubulin, and intermediate filaments [66, 67]. Engineered bacterial strains with optimized morphology demonstrated significant advantages, including accelerated cell proliferation, high-density cultivation, simplified downstream processing, and enhanced accumulation of inclusion bodies [68–70]. Compared with the control strain EcCon, the *b12fla*-overexpressing strain EcFHP exhibited significant morphological alterations, characterized by obvious increase in cellular width (Fig. 4A and B). Cells of the mutant strain exhibited several distinct morphological features: certain cells became broader at only one end, assuming a gourd-like (calabash) profile; others showed became broader in the middle of the cell, presenting a spindle-shaped morphology. While others became broader at two ends, resulting in a dumbbell-like appearance (Fig. 4A and B). Additionally, SEM identified cells exhibiting significantly reduced cell length and increased width, exhibiting spherical or ellipsoidal morphologies. Systematic and in-depth investigations about the regulatory networks will be conducted in follow-up studies. Nevertheless, In addition, SEM analysis was performed for E. adhaerens strains (Ea-WT and Ea-b12fla and Ea-∆b12 ), the results were shown in figure S9. The results showed that neither overexpression nor knockout of *b12fla* gene caused any apparent morphological changes in the host strain *E. adhaeren*s.

### Effects of FHP expressing on growth and VB_12_ production of *E. adhaerens*

In industrial production, VB_12_ is predominantly biosynthesized through aerobic fermentation by *E. adhaerens*. Previous studies demonstrated that oxygen restriction leads to significant upregulation of flavohemoglobin in *E. adhaerens*, concomitantly inducing upregulation of genes in heme biosynthesis pathway due to increased cofactor demand of *b12fla*. In this study, to evaluate the impact of FHP expression on biomass, oxygen utilization, and VB₁₂ production of *E. adhaerens*, we constructed a *b12fla*-overexpressing strain Ea-b12fla. A knockout strain Ea-∆b12fla was constructed using a homologous recombination single-crossover strategy in our previous study [67].

**Fig. 5:**
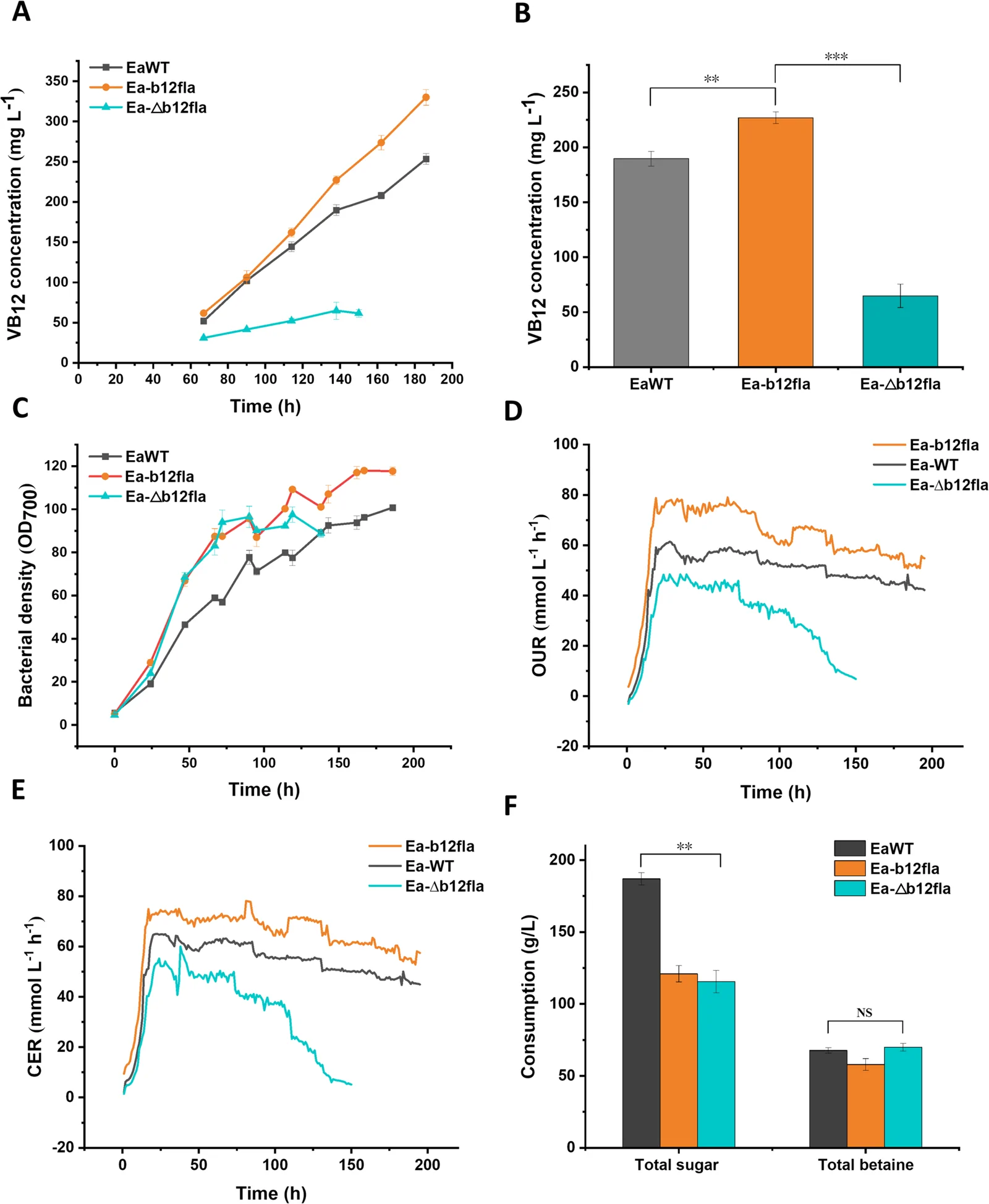
Effect of *b12fla* on VB_12_ biosynthesis and physiological metabolism in *E. adhaerens*. **A** Vitamin B_12_ production, **B** Vitamin B_12_ production at the 138th h, **C** OD_700_ of Ea-b12fla, Ea-∆b12fla and Ea-WT strain in 50-L bioreactor. **D** OUR, **E** CER of Ea-b12fla, Ea-∆b12fla and Ea-WT strain in 50-L bioreactor. **F** Consumption of sugar and betaine of Ea-b12fla, Ea-∆b12fla and Ea-WT strain in 50-L bioreactor. Data are presented as mean values of three biological replicates ± standard deviation. Asterisks depict significant differences between samples: *p < 0.05, **p < 0.01 and ***p < 0.001

Fermentation in a 50-L bioreactor was conducted to investigate FHP’s impact on *E. adhaerens* physiology and VB₁₂ production. The results revealed that the VB_12_ production of the knock-out strain presented a downward trend after 138 h (Fig. 5A), the VB_12_ concentration reached 61.5 mg L^− 1^ at the 150th hour, which was lower than that at the 138th hour (64.9 mg L^− 1^). Concomitantly, a rapid increase in pH was observed in the fermentation broth, reaching pH 8.16 at the138th hour (Fig S10). We further compared the VB_12_ production at the 138th hour of fermentation, the VB_12_ production of Ea-∆b12fla was 64.9 mg L^− 1^, which decreased by approximately 70.9% and 77.0%, respectively, compared with that of Ea-WT and Ea-b12fla (Fig. 5B). This result indicates that the deletion of the *fla* gene exerts a strong inhibitory effect on the respiratory metabolism of *Ensifer adhaerens*. At the end of 186-hour fermentation, the maximum VB_12_ production of Ea-b12fla reached 323 mg L^− 1^, which was 29.9% higher than that of the wild-type strain Ea-WT (248.7 mg L^− 1^). This confirms that the *b12fla* gene plays a significant promoting role in the respiratory metabolism of the *Ensifer adhaerens* The OD_700_ of overexpressing strain reached 117, which was 11.7% higher than that of WT strains (Fig. 5C). This growth-promoting effect aligns with observations in *E. coli* overexpressing *b12fla*, confirming an evolutionarily conserved role for flavohemoproteins in enhancing bacterial growth under oxidative stress. Interestingly, during the early fermentation phase, the knockout strain exhibited enhanced growth compared to wild-type strain (Fig. 5C). The underlying mechanisms require in-depth exploration in future studies.

### Effects of FHP expressing on metabolism of *E. adhaerens*

Oxygen uptake rate (OUR) and Carbon-dioxide Escape Rate (CER) were further evaluated in a 50-L bioreactor. The OUR profiles showed that both the wild-type strain and the Ea-b12fla strain could maintain relatively stable oxygen uptake rate and carbon dioxide evolution rate. After 24 h of fermentation, the OUR of all strains peaked, the OUR of the ‌FHP-overexpressing strain Ea-b12fla‌ reached 75 mol L^−1^ h^−1^, which was 27% higher than that of the wild-type strain, Moreover, throughout the remainder of the fermentation cycle (post-24 h), the OUR of Ea-b12fla remained persistently higher than that of the wild-type strain, consistently exceeding it by over 20% (Fig. 5D). Conversely, the knock-out strain‌ Ea-∆b12fla exhibited a significant reduction in OUR (only 48 mol L^−1^ h^− 1^). Simultaneously, it was observed that the oxygen uptake rate (OUR) of Ea-∆b12fla strain underwent a sharp decline after approximately 70 h, whereas the wild-type and overexpression strains exhibited a gradual decrease (Fig. 5D). This indicates that FHP plays a critical role in mediating cellular oxygen uptake for growth during the later stages of cultivation. These results ‌demonstrate that the *b12fla* gene is crucial for‌ oxygen uptake and utilization in *E. adhaeren*. Overexpression of the flavohemoprotein (FHP) ‌enables‌ cells to achieve faster growth rates under specific oxygen conditions, thereby attaining‌ higher biomass. Similar to the trend in the OUR, strain Ea-∆b12fla consistently exhibited approximately > 20% higher CER than the wild-type throughout the cultivation (after 24 h) (Fig. 5E). In contrast, the Ea-∆b12fla strain demonstrated a significant reduction in CER, with a notably pronounced decline observed around 70 h. These results indicated that FHP plays a pivotal role in modulating respiratory metabolism. Overexpression of FHP substantially augments respiratory capacity, consequently accelerating cellular growth. Thus, overexpression of FHP represents an economical and industrially applicable strategy for addressing oxygen utilization challenges in high-cell-density fermentation.

The consumption of total sugar was 117 g L^− 1^ in strain Ea-b12fla, which was 57.2% lower than that of wild-type strain (184 g L^− 1^) (Fig. 5F). The Ea-b12fla strain exhibited significantly enhanced vitamin B_12_ yield coefficient to glucose consumption: a 104% improvement in sugar conversion (2.76 and 1.35 mg g^− 1^ for *vb12fla* strain and WT strain respectively) and a 36.4% increase in betaine conversion (5.32 and 3.90 mg g^− 1^ for Ea-b12fla strain and wild-type strain respectively) (Fig. 5A and F). We supposed that over expression of FHP increase in demand for heme, which consequently elevates flux the porphyrin metabolic pathway, thereby redirecting sugar substrates toward VB₁₂ biosynthesis. In summary, *b12fla* overexpression enhances *E. adhaerens* growth and VB₁₂ production while significantly improving glucose and betaine conversion efficiencies, potentially resolving the long-standing bottleneck of low substrate conversion in industrial VB₁₂ fermentation; conversely, *b12fla* knockout severely reduced VB₁₂ biosynthesis.

## Conclusion

A novel flavohemoglobin FHP from *E. adhaerens* were successfully characterized and proven to possess high NADH oxidase activity. The expression of FHP in *E. coli* and *E. adhaerens* promotes the cell growth, cell metabolism and carbon source utilization. Overexpression of the *b12fla* gene in *E. adhaerens* greatly enhanced strain to overproduce VB_12_, during the 50-L fermentation, the wild type strain produced only 248.7 mg L^− 1^ VB_12_ whereas the over-expression strain yielded 323 mg L^− 1^ VB_12_. Simultaneously, cell growth, cell metabolism and sugar utilization of *E. adhaerens* were also obviously enhanced by overexpression of the *b12fla*. Additionally, morphological alterations were observed in flavohemoglobin-overexpressing strains. ‌Collectively, these findings demonstrate that flavohemoglobin (FHP) enhances cellular oxygen utilization while simultaneously improving the assimilation efficiency of carbon and nitrogen sources, and reveal the potential of flavohemoglobin for applications in high-density fermentation processes.

## Supplementary Information

Below is the link to the electronic supplementary material.

Supplementary Material 1.

## Data Availability

No datasets were generated or analysed during the current study.
