# Supplementary material for: Identification, functional characterization of a novel flavohemoglobin from Ensifer adhaerens and its application to enhancing vitamin B12 production
Source: Microb Cell Fact. 2026 Jul 24;25:219. doi: 10.1186/s12934-026-03073-z (PMC13685156; doi:10.1186/s12934-026-03073-z)
Supplement: Supplementary file 1 [file 12934_2026_3073_MOESM1_ESM.docx]

**Supplementary Material**

**Identification, functional characterization of a novel flavohemoglobin from *Ensifer adhaerens* and its application to enhancing vitamin B_12_ production**

Jinmei Zhu ^1^, Shiyu Wang ^1^, Bo Li ^2^, Bolun Wang ^1^, Ping Wang ^2^, Jiequn Wu ^1*^, Zejian Wang ^2*^

^1^ Collaborative Innovation Center of Yangtze River Delta Region Green Pharmaceuticals, Zhejiang University of Technology, Hangzhou 310014, China

^2^ State Key Laboratory of Bioreactor Engineering, East China University of Science and Technology, Shanghai, China

^*^ Correspondence: Zejian Wang ([wzjvictory@163.com](mailto:wzjvictory@163.com)) and Jiequn Wu (jiequnwu@zjut.edu.cn)

**Table of Contents**

**Supplementary Materials and methods**

1. Sequence Alignment and Phylogenetic Tree Construction
2. Bacterial cell enumeration using flow cytometry
3. Determination of intracellular ATP concentration in *Escherichia coli*
4. Quantification of intracellular NAD⁺and NADH levels in *Escherichia coli*
5. Identification of heme cofactor bound to FHP
6. HPLC analysis of flavin cofactor bound to FHP and HMP

**Supplementary Figures**

Figure S1. Phylogenetic analysis of 200 candidate flavohemoglobins (NCBI)

Figure S2. The characteristic spectra of heme and FHP

Figure S3. HPLC analysis of flavin cofactor bound to FHP and HMP

Figure S4.SDS-PAGE of purified proteins

Figure S5.Determination of kinetic constants for flavohemoglobin reactions

Figure S6. Number of particles of EcCon and EcFHP strain.

Figure S7 Residual ammonia concentration in culture media

Figure S8. Yield of biomass on ammonia nitrogen (Y_OD/NH₃-N_, g^-1^) of strains

Figure S9. Morphology FHP-overexpression *E. adhaerens* strain the wild-type strain

Figure S10. pH of Ea-b12fla, Ea-∆b12fla and Ea-WT strain in 50-L bioreactor.

**Supplementary Tables**

Table S1. Strains and plasmids used in this study.

Table S2. Primers used in this study.

Table S3. Sequence of genes and proteins

**Supplementary References**

**Supplementary Materials and methods**

**1.** Sequence Alignment and Phylogenetic Tree Construction

The amino acid sequence of FHP was used as a query to perform a BLASTP search against the non-redundant protein sequence database on the NCBI website (https://blast.ncbi.nlm.nih.gov/), with the maximum number of target sequences set to 1000 and all other parameters maintained as default. Sequences sharing 30–80% identity with the query were retrieved in FASTA format, and 200 sequences were selected, among which 10 have been reported.Multiple sequence alignment was carried out using ClustalW integrated in MEGA 12.0. A neighbor-joining tree was constructed with 1000 bootstrap replicates to assess branch support and all other parameters maintained as default. The phylogenetic tree was visualized using the Interactive Tree of Life (iTOL) platform (https://itol.embl.de).

**2. Bacterial cell enumeration using flow cytometry**

A CytoFLEX flow cytometer (Beckman, USA) was utilized to count the *E. coli*. Three colonies were picked and incubated in 5 mL LB medium containing 50 μg mL^-1^ kanamycin and cultured overnight at 37 °C with shaking at 220 rpm. The overnight cultures were used as seed cultures for 5 mL fresh LB media containing 50 μg mL^-1^ kanamycin and incubated at 37 °C until the OD_600_ reached 0.6. The cultures were induced with 0.1 mM isopropyl-β-D-thiogalactopyranoside (IPTG) at 37 °C for 4 h. A 200 μL aliquot was taken from each culture, washed twice with PBS. The samples were injected into the CytoFLEX cytometer, with the injection speed controlled to allow 1000-2000 cells per second to pass through. Each sample was injected for a duration of 30 seconds.

3. **Determination of intracellular ATP concentration in *Escherichia coli***

A stock ATP standard solution (0.5 mM) was prepared and serially diluted with the lysis buffer provided in the enhanced ATP assay kit to generate a series of gradient standard samples at final concentrations of 0, 0.5, 1, 2 and 3 μM. ATP contents were quantified using the Beyotime enhanced ATP detection kit, and detailed experimental procedures were performed in accordance with the manufacturer’s instructions; the corresponding standard calibration curve was obtained.

For bacterial sample preparation, *E. coli* cells were inoculated into LB medium at an inoculation ratio of 1% (v/v). After incubation until OD_600_ reached 0.6 - 0.8, isopropyl β-D-1-thiogalactopyranoside (IPTG) was added to a final concentration of 0.2 mM for target protein induction. A total of 2 mL bacterial suspension was harvested at 4 h, 8 h and 20 h post-induction, respectively. Cell pellets were collected by centrifugation at 12,000×g for 2 min, discarded the supernatant, and rinsed twice with pre-cooled PBS prior to subsequent ATP measurement following the kit’s protocol. Black 96-well plates were used for luminescence detection as required by the chemiluminescence-based assay principle.

**4. Quantification of intracellular NAD⁺and NADH levels in *Escherichia coli***

A 25 μM NADH stock standard solution was prepared and diluted with the NAD^+^/NADH extraction buffer included in the assay kit to obtain standard samples with final amounts of 0, 62.5, 125, 500, 1000, 2000 and 4000 pmol. The contents of NADH were determined with the Beyotime NAD^+^/NADH Quantification Kit (WST-8 colorimetric method) to obtain the standard curve .

*E coli* strains were cultured in LB medium with 1% (v/v) inoculation. When OD_600_ was adjusted to 0.6-0.8, IPTG was supplemented at a final concentration of 0.2 mM to induce FHP expression. Six milliliters of bacterial culture were collected at 4 h after induction. Cells were pelleted via centrifugation at 12,000×g for 2 min, the supernatant was discarded, and cell pellets were washed twice with pre-cooled PBS before sample extraction. All NAD^+^ and NADH quantification operations were conducted according to the kit specification. Given the high susceptibility of NADH to degradation, all experimental manipulations were completed rapidly under dark conditions.

**5. Identification of heme cofactor bound to FHP**

Identification of the heme cofactor was performed according to the previously reported literature method^[1]^. For spectroscopic analysis of heme cofactor, a 40 μM heme standard solution was prepared in 100 mM PBS (pH 7.5). The ultraviolet-visible (UV-Vis) absorbance spectrum scanning from 190 nm to 500 nm using a UV-1800 UV spectrophotometer (SHIMADZU, Japan), where free heme exhibited a characteristic absorption maximum at about 380 nm. For protein samples, 100 μL of purified FHP protein (8.5 mg mL^-1^) was added into 900 μL of 100 mM PBS (pH 7.5), followed by UV-Vis spectral scanning over the wavelength range of 190-500 nm.

**6. HPLC analysis of flavin cofactor bound to FHP and HMP**

HPLC analysis of flavin cofactor was performed according to the previously reported literature method^[2]^.High-performance liquid chromatography (HPLC) analysis was performed on an Agilent 1200 series HPLC system equipped with an Elite SinoChrom ODS-BP C18 column (5 μm, 4.6*250mm) at a flow rate of 0.8 mL min^-1^. Mobile phase A was 5 mM ammonium acetate buffer (pH 6.0).. Mobile phase A and mobile phase B (methanol) were used with gradient: 0-3 min 15% B, 3-23 min 15-75% B, 23-25 min 75% B, 25-30 min 75-15% B, 30-32 min 15% B. A diode array detector (DAD) was used to monitor flavin compounds at a detection wavelength of 264 nm. Additionally, authentic flavin adenine dinucleotide (FAD) and flavin mononucleotide (FMN) were used as standards for HPLC retention time.

**Figure S1.Phylogenetic analysis of 200 candidate flavohemoglobins (NCBI) with 30-80% amino acid sequence identity to FHP**

**Figure S2. The characteristic spectra of heme and FHP**

Free heme (dark gray trace) exhibited a typical broad Soret band at ~380 nm, while the absorbance maximum of the flavohemoglobin FHP (orange trace) was located at ~405 nm in PBS buffer.

**Figure S3. HPLC analysis of flavin cofactor bound to FHP and HMP**

Chromatograms of authentic FAD, authentic FMN, and the flavin cofactor isolated from heat-denatured FHP and HMP are monitored at 264 nm. The commercially available FMN standard used in this study only has a purity of approximately 80%, and impurity peaks were observed in its corresponding HPLC chromatogram.

**Figure S4. SDS-PAGE of purified VHb (A), FHP and HMP (B)**

**Figure S5. Determination of kinetic constants for FHP(A) and HMP(B) reaction**

**Figure S6. Number of particles of EcCon and EcFHP strain**.

**Figure S7 Residual ammonia concentration in culture media**

**Figure S8. Yield of biomass on ammonia nitrogen (Y_OD/NH₃-N_, g^-1^) of strain EcFHP, EcVHb, EcHPM and EcCon.** Data are presented as mean values of three biological replicates ±standard deviation. *p < 0.05, **p < 0.01 and ***p < 0.001.

**Figure S9. Morphology FHP-overexpression *E. adhaerens* strain the wild-type strain.** Scanning electron microscopy studies on morphology of EaWT (A), Ea-∆b12fla (B), Ea-b12fla (C) and (D).

**Figure S10. pH of Ea-b12fla,** **Ea-∆b12fla and Ea-WT strain in 50-L bioreactor.**

**Table S1. Strains and plasmids used in this study.**

|  | Description | Reference or source |
| --- | --- | --- |
| **Strains** |  |  |
| *E. coli* DH5α | TreliefTtMm 5α Chemically Competent Cell | TsingKe |
| *E. coli* BL21 (DE3) | Expression Chemically Competent Cell | TsingKe |
| EcCon | *E. coli* BL21 (DE3) carrying vector pET28a | This study |
| EcFHP | *E. coli* BL21 (DE3) carrying vector pET28a-*b12fla* | This study |
| EcVHb | *E. coli* BL21 (DE3) carrying vector pET28a-*vgb* | This study |
| EcHMP | *E. coli* BL21 (DE3) carrying vector pET28a-*hmp* | This study |
| EcFHP-ΔrecA | *E. coli* BL21 (DE3) with b12fla integration into the recA region | This study |
| EcFHP-ara | *E. coli* BL21 (DE3) with b12fla integration into the ara region | This study |
| Ea-WT | *Ensifer adhaerens* Casida A for roduction of vitamin B12 | [3] |
| Ea-b12fla | Strain of *Ensifer adhaerens* Casida A overexpression *b12fla* | This study |
| Ea-∆b12fla | Strain of *Ensifer adhaerens* Casida A knock-out *b12fla* | [3] |
| **Plasmids** |  |  |
| pET28a | *E. coli* expression vector (Kan, T7 promoter) | This study |
| pET28a-*vgb* | pET28a carrying gene *vgb* from *Vitreoscilla* fused with His tag in C-terminus | This study |
| pET28a-*b12fla* | pET28a carrying gene *b12fla* from *Ensifer adhaerens* Casida A fused with His tag in C-terminus | This study |
| pET28a-*hmpBh* | pET28a carrying gene *hmpBh* from *Halalkalibacterium halodurans* fused with His tag in C-terminus | This study |
| pEcCas | Constitutive expression of cas9 and inducible expression of lambda RED and sgRNA | Plasmid #73227 [4] |
| pEcgRNA-sg^recA^-L^RecA^-*b12fla*- R^RecA^ | pEcgRNA carrying sg^recA^ and the upstream and downstream homologous arms of gene *b12fla* from *Ensifer adhaerens* Casida A | This study |
| pEcgRNA-sg^ara^-L^ara^-*b12fla*- R^ara^ | pEcgRNA carrying sg^ara^ and the upstream and downstream homologous arms of gene *b12fla* from *Ensifer adhaerens* Casida A | This study |
| pOJ260-*b12fla* | pOJ260 carrying sg^recA^ and the upstream and downstream homologous arms of gene *b12fla* from *Ensifer adhaerens* Casida A | This study |
| pOJ260-∆*b12fla* | pOJ260 carrying sg^recA^ and the upstream and downstream homologous arms of gene *b12fla* from *Ensifer adhaerens* Casida A | This study |

**Table S2. Primers and sequence used in this study.**

| Name | Sequence (5′-3′) | Description |
| --- | --- | --- |
| 28a-*b12fla*-F | CTTTAAGAAGGAGATATACCATGCATCACCACCACCACCATAATT | Amplification of the *b2fla* gene for ligation into plasmid pET28a |
| 28a-*b12fla*-R | TCGAGTGCGGCCGCAAGCTTTTACGCGGCAATCAATTCATCTGCC |  |
| 28a-*vgb*-F | CTTTAAGAAGGAGATATACCATGCATCACCATCACCACCACCTAG | Amplification of the *vgb* gene for ligation into plasmid pET28a |
| 28a-*vgb*-R | TCGAGTGCGGCCGCAAGCTTTTACTCGACCGCTTGTGCATACAGG |  |
| Sg^recA^-F | AGTGCCTGCTCGCCGGTGTCGTTTTAGAGCTAGAAATAGC | Amplification of the Sg^recA^ |
| Sg^recA^-R | GACACCGGCGAGCAGGCACTACTAGTATTATACCTAGGAC |  |
| Sg^ara^-F | CCAGGTGTCAGTGAACAGCGGTTTTAGAGCTAGAAATAGC | Amplification of the Sg^ara^ |
| Sg^ara^-R | CGCTGTTCACTGACACCTGGACTAGTATTATACCTAGGAC |  |
| L^recA^ | GTTCATGTGCAGCTCCATAACTGGATCTTAACTTTTTCTG | homologous arm used for recombination in the recA region |
| R^recA^ | AACTTATCATCCCCTTTTGCAAAGTGATTACCGATATTGC |  |
| L^ara^ | GTTCATGTGCAGCTCCATAACAGCAAAAATCGCCGCAC | homologous arm used for recombination in the ara region |
| R^ara^ | AACTTATCATCCCCTTTTGCCGCTTCAGCCATACTTTTCA |  |
| ProP-F | CGACGGCCAGTGCCAAGCTTTTGTCCCAAATAGCCCCATG | Amplification of the promoter of ORF WFP91548.1 |
| ProP-R | AAATGCAGCCGGCAATTCATTTCTGACCTCCAGTCAAAAG |  |
| 260-*b12fla*-F | ATGAATTGCCGGCTGCATTT | Amplification of the *b2fla* gene for ligation into plasmid pOJ260 |
| 260-*b12fla*-R | TATGACATGATTACGAATTCTCAGGCTGCAATGAGTTCGT |  |

**Table S3. Sequence of genes and proteins**

| Name | Sequence |
| --- | --- |
| *b12fla* | AATTGTAGGCTCCATTTTCAAGATCACCAACCCGCTCCGACCGATACCTCTCACCGCAAAGAAAAAATCATGCCGAAGCCGCTGAGCCCGGAAACGGTACAGATTGTGAAGGCGTCCGCGCCGGCCCTGGCGGCGCACGGCACCGAAATCACGAAAAGCATGTATGCACGTCTGTTCCGCGACGACCACATCCGTGCGCTGTTTAATCATGCGAACCAGGGTGAGGGTGGCTCTCAGGTTAATGCCCTTGCTGCGGCGATTCTGGCGTACGCTCAAAACATTGACAACTTGGGCGTGCTGGGCCCAGTGGTGGAACGTATTGCCCACAAACATATTGGTTATCATATCCTTCCGGAGCACTACCCGTTTGTAGCCAAGGCCTTGTTCGCCGCGGTTGAAGATGTCCTAGGCAGCGCTGCTTCTCCGGAGTTGCTGAACGCATGGGGTGAAGCTTACTGGTTTTTGGCTGACATTCTGAAAGAGCGGGAGACGGCGATCCGCGGTGAATTAATGGAACGTAGCGGTGGATGGAGCGGCTGGCGTGACTTTGTTATTGCATCGAAAGTGCGCGAGTCCGATGTGGTCACCAGCTTCATTCTGAAGCCGAAGGACGGCGGCCGTGTCGCGCGCCACCGTCCGGGTCAATATCTGACCTTTCGTTTCCAACTGGATAACGGCCTGGAGATGAAACGTAATTATTCCATCTCCAGCGGTCCAAACGACAGCTTCTACCGCATCAGTGTTAAGCGCGAGGCGCAGGGCCAGGGTGGTTCTCGTTACCTGCACGATCAGGCAACCGTGGGTACTGTCCTTAGCGCGACCCCTCCGGCGGGTGATTTCTTCCTGCCGGAGGAACCAGAGGCGCCAGTTGTTCTGCTGTCAGGCGGCGTGGGCTTGACCCCGATGGTTTCCATCTTAGAGACCATCGCCGAAGAACACCCGAATCTGGAAGCGCATTTTGTGCATGGCGCACTCAACAGCGGCACCCATGCAATGGATAGACACGTGCGTGAGCTGGCAGACGCTCACGGTAAAATCACGATCTCGAGCTTCTACAGCGCTCCGGCAAATGGCGACGCTGTTGGTCTGTCTCACGACTTCGATGGTTTTATCACCGCGGAGTGGCTGCGTGAGCATACTCCGATTGCAAACGCAGAGTTCTACCTGTGCGGTCCGAAGCCGTTCCTGCGCGCGTTGGTTCCGGCGCTGGCGCGTGAAGGTGTGAGCAGCGAACGTGTTCATTATGAATTTTTTGGTCCGGCAGATGAATTGATTGCCGCGTAA |
| FHP | NCRLHFQDHQPAPTDTSHRKEKIMPKPLSPETVQIVKASAPALAAHGTEITKSMYARLFRDDHIRALFNHANQGEGGSQVNALAAAILAYAQNIDNLGVLGPVVERIAHKHIGYHILPEHYPFVAKALFAAVEDVLGSAASPELLNAWGEAYWFLADILKERETAIRGELMERSGGWSGWRDFVIASKVRESDVVTSFILKPKDGGRVARHRPGQYLTFRFQLDNGLEMKRNYSISSGPNDSFYRISVKREAQGQGGSRYLHDQATVGTVLSATPPAGDFFLPEEPEAPVVLLSGGVGLTPMVSILETIAEEHPNLEAHFVHGALNSGTHAMDRHVRELADAHGKITISSFYSAPANGDAVGLSHDFDGFITAEWLREHTPIANAEFYLCGPKPFLRALVPALAREGVSSERVHYEFFGPADELIAA |
| ProP | TTGTCCCAAATAGCCCCATGTCCTTTGCGCCCCGCCCTTCGAGCGAGCATTTGGTTTCGGCTATCTCTCGACTGAATTTCATTTATTGTGGTTCGTGAGCCGTTGCGGCAGTCGTCGCCAGGGGCGCCCGTGCATTCTTGGTGCTGCGCGGTGCTGATCTTTGCATCGTTCAGAATCACCTGGTCGGGGCGTTTGCCGAATCTCTGCGTTGATTCTGCTGATTCCTCAGGCCGCCATCCTTTCTCCACCAAGCGCTTTCTTGGAGCGAATGCTGTTTTAGGCATTCACTGGACATCTGAAACGGGGTTCGCGTCCAGCTTTTCCCCTGTTTTGGGCGGGTTTCTGCCTCTTTCTGAAACAGGCGACCCCCACCTTCCTTATGCTAACCCCCCGTCGCTAAAGGGTTTTCTTAATGCTTTGTTAATTTTCTGCGGGGGTCTGCGGCGATTTTGTGTCGTTTCAGCAACAGGCCTCGGCGAAGGGCGATGCAAAGGACGTGAACTCGCTAGTTGGACGTTGCGCCGGTGATCCGGTCTTGTATTTTCAACTCCGGAAGCAAGGGCGGTTGATCGTCCGACTTCTTGAACGTTGGGGATGGGGCAGGGAAAGCTGTCCTTCAGCAAAATACCCAGACCCGTTTGAAACTTTTGACTGGAGGTCAGAA |

**3. Supplementary References**

[1] Lewis JA., Escalante-Semerena JC. 2006. The FAD-dependent tricarballylate dehydrogenase (TcuA) enzyme of *Salmonella enterica* converts tricarballylate into *cis*-aconitate. J Bacteriol. 188(15): 5479-5486.

[2] Hopp MT, Vaidya SM, Grimmig KM, Strudthoff LJ, Clauser JC, Yuan X, Singh S, Müller J, Oldenburg J, Hamza I, Imhof D. 2024. Quantitative analysis of heme and hemoglobin for the detection of intravascular hemolysis. Anal Chim Acta. 1312: 342766.

[3] Li B, Chen X, Zhao D, Liu Z, Li J, Siddique MS, Wu J, Zhuang Y, Wang Z. 2024. Physiological Metabolic Analysis of VB12 Accumulation in *Ensifer adhaerens Casida A* Enhanced by Oxygen Limitation. Biotechnol J. 19(9): e202400305.

[4] Jiang Y, Chen B, Duan C, Sun B, Yang J, Yang S. 2015. Multigene editing in the *Escherichia coli* genome via the CRISPR-Cas9 system. Appl Environ Microbiol. 81(7): 2506-2514.
